# The Aftermath of Obstruction: Decoding Collateral Pathways in Superior Vena Cava Syndrome

**DOI:** 10.7759/cureus.83078

**Published:** 2025-04-27

**Authors:** Schafer Paladichuk, Benjamin Soderling, Montana Hawksford, Sage Sorensen, Natalie Yoshioka, Christian Heck

**Affiliations:** 1 Department of Anatomy, Pacific Northwest University of Health Sciences, Yakima, USA

**Keywords:** cardiovascular, collateral venous formation, mediastinum, superior vena cava syndrome, vena cava

## Abstract

Superior vena cava (SVC) syndrome (SVCS) is any occlusion of the SVC, resulting in venous blood backup. Common causes of SVCS include mediastinal tumors and, less frequently, implanted venous devices. Understanding collateral venous formation in SVCS is crucial for identifying alternative pathways for venous return and alleviating symptoms of venous obstruction. The SVC, formed by the anastomosis of the right and left brachiocephalic veins, drains blood from structures above the diaphragm into the right atrium. Here, we provide a unique postmortem analysis of SVCS and collateral venous formation to support clinical identification and treatment.

A 48-year-old cadaver with a limited medical history of metastatic rectal cancer was dissected in an anatomy course. The SVC was observed to be occluded in its entirety. The likely cause of SVCS was mediastinal tumors. Vessel dilation was observed within the azygos system, intercostal, right thoracic-epigastric, inferior epigastric, and internal thoracic veins. Collateral venous formation was found by way of the azygos and hemiazygos veins, which joined inferiorly to form a single vessel draining into the inferior vena cava (IVC), and multiple left intercostal veins formed single communications with the azygos vein.

Recognizing this complex anatomy and patterns of the azygos venous system is crucial in understanding alternative blood flow pathways in cases of SVCS. Variations in this system can form critical venous collaterals, bypassing obstructions and relieving venous congestion. Although visualizing these collateral networks on imaging can be challenging, postmortem gross findings offer crucial insights into collateral formations and potential patterns in SVCS presentation. Ultimately, detailed understanding and identification of azygos venous patterns improve patient safety, support accurate diagnosis, and optimize treatment outcomes across a range of medical and surgical disciplines.

## Introduction

The superior vena cava (SVC) is a large and significant upper-body vein responsible for carrying deoxygenated blood directly to the right atrium of the heart [1,2]. SVC syndrome (SVCS) results from any condition causing obstruction of blood flow through the SVC, resulting in symptoms ranging from benign to life-threatening [1,3-5]. Furthermore, obstruction of the SVC can result in collateral venous formation redirecting deoxygenated blood through tributaries, such as the azygos system, to the inferior vena cava (IVC) and ultimately into the heart [6]. Postmortem descriptions of SVCS are currently lacking despite this broad clinical significance. Here, we present a cadaveric case study of SVCS and the resultant collateral venous formation.

The SVC is formed from the union of the right and left brachiocephalic veins at the level of the first intercostal space. The SVC receives venous drainage from the majority of structures superior to the diaphragm and transports this drainage to the right atrium of the heart [2]. Conversely, the IVC is responsible for draining structures inferior to the diaphragm directly into the right atrium of the heart [1,2]. Typically, both the SVC and IVC communicate with the azygos system of veins, a complex of valveless veins along the posterior thoracic and abdominal walls [2].

The azygos system of veins drains regions of the back, thoracic wall, abdominal wall, and thoracic viscera. Major components of this system include the azygos vein, the hemiazygos vein, and the accessory hemiazygos vein, although the branching patterns of this system are highly variable [7,8]. The azygos vein is a unilateral vessel ascending along the posterior thoracic wall, receiving deoxygenated blood from the hemiazygos vein, accessory hemiazygos vein, and intercostal veins. The azygos vein drains directly into the SVC at the level of the right second intercostal space with an additional communication inferiorly with the IVC [5]. The hemiazygos vein drains blood from the left posterior mediastinum below the vertebral level of T8, and the accessory hemiazygos vein drains blood from the posterior mediastinum above the vertebral level of T8 [5]. The azygos venous system serves as an important pathway for decompression of the SVC when there is intraluminal blockage or external compression [1,5].

The clinical presentation of SVCS varies widely and depends on the severity, speed, and location of the obstruction [2]. The location of the SVC obstruction has a strong influence on symptoms and the resulting collateral venous formation [9,10]. More than 80% of SVCS arise from malignant mediastinal tumors, of which 75%-80% are due to bronchogenic tumors, with most being small-cell carcinoma of the lung [1,3,4,11,12]. A secondary benign potential cause of SVCS is a sequelae of poorly positioned implantable venous devices (central venous catheter, cardiac pacemaker wire, etc.) [13]. This case report aims to (1) describe the location and severity of the SVC obstruction using SVCS classifications developed by Standford et al. [10] and Azizi et al. [14] and (2) describe resultant collateral venous formations. Although rare, postmortem descriptions of SVCS sequelae are important contributions to the documentation and classification of venous remodeling, which in turn influences therapy.

## Case presentation

**Figure 1:**
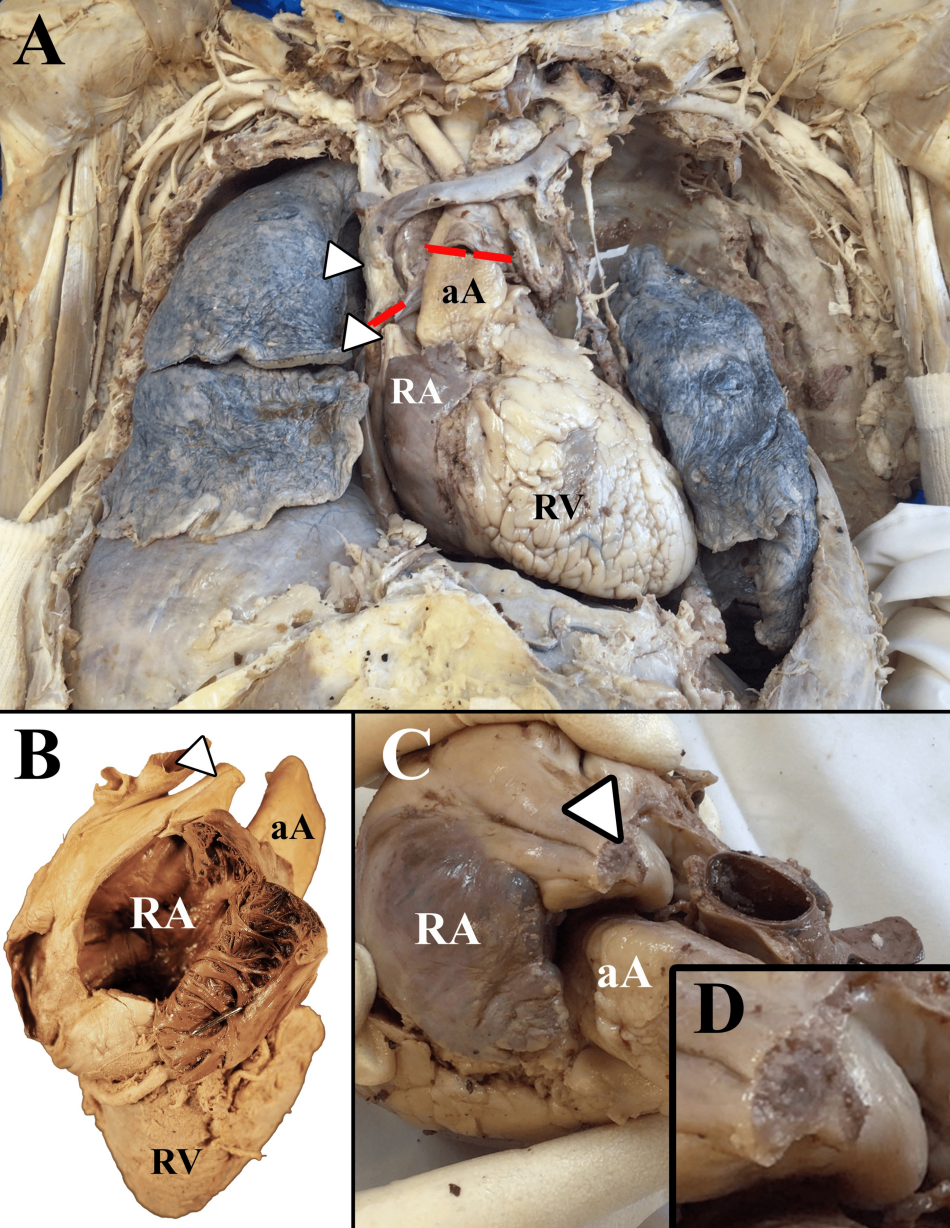
Gross images of the cadaver with superior vena cava syndrome. (A) Anterior view of the thoracic cavity. (B) Supero-anterior view of the heart with the right atrium (RA) opened. The superior vena cava (arrowhead) narrows distal to the opening into the right atrium. (C) Superior view of the heart and roots of great vessels including the superior vena cava (arrowhead). (D) Magnified view of the completely occluded superior vena cava. Arrowheads: superior vena cava; red lines: dissection cuts; aA: ascending aorta; RV: right ventricle

**Figure 2:**
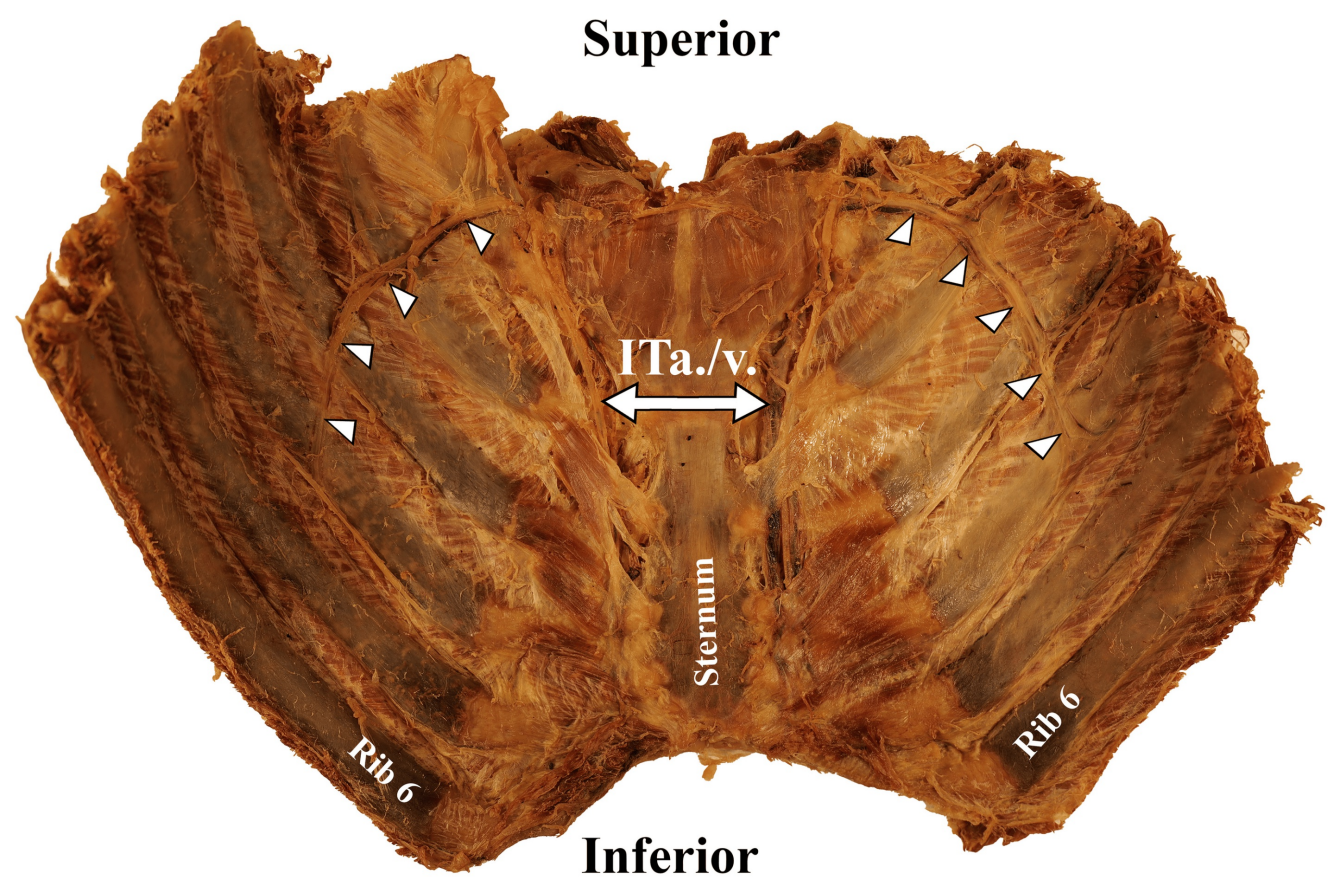
Posterior view of anterior thoracic cage (ribs 1-6 bilaterally). Bilateral collateral vascular bundles (arrowheads) branch off the internal thoracic artery and vein (ITa./v.).

SVCS was discovered in a 48-year-old donor body cadaver during routine thoracic dissection for a university clinical anatomy course. The rights of the donor were protected, and past medical history was limited to the cause of death: metastatic rectal cancer. The donor cadaver did not have any observable previous surgeries in the thoracic region. To secure an appropriate view of thoracic vasculature, the anterior chest wall was removed, lungs were resected at the pulmonary root, and adjacent vasculature was dissected (Figures 1, 2). The heart was removed by severing the great vessels approximately 2 cm distal to the heart (Figure 1). The posterior thoracic and abdominal walls were thoroughly dissected upon removal of the viscera.

**Figure 3:**
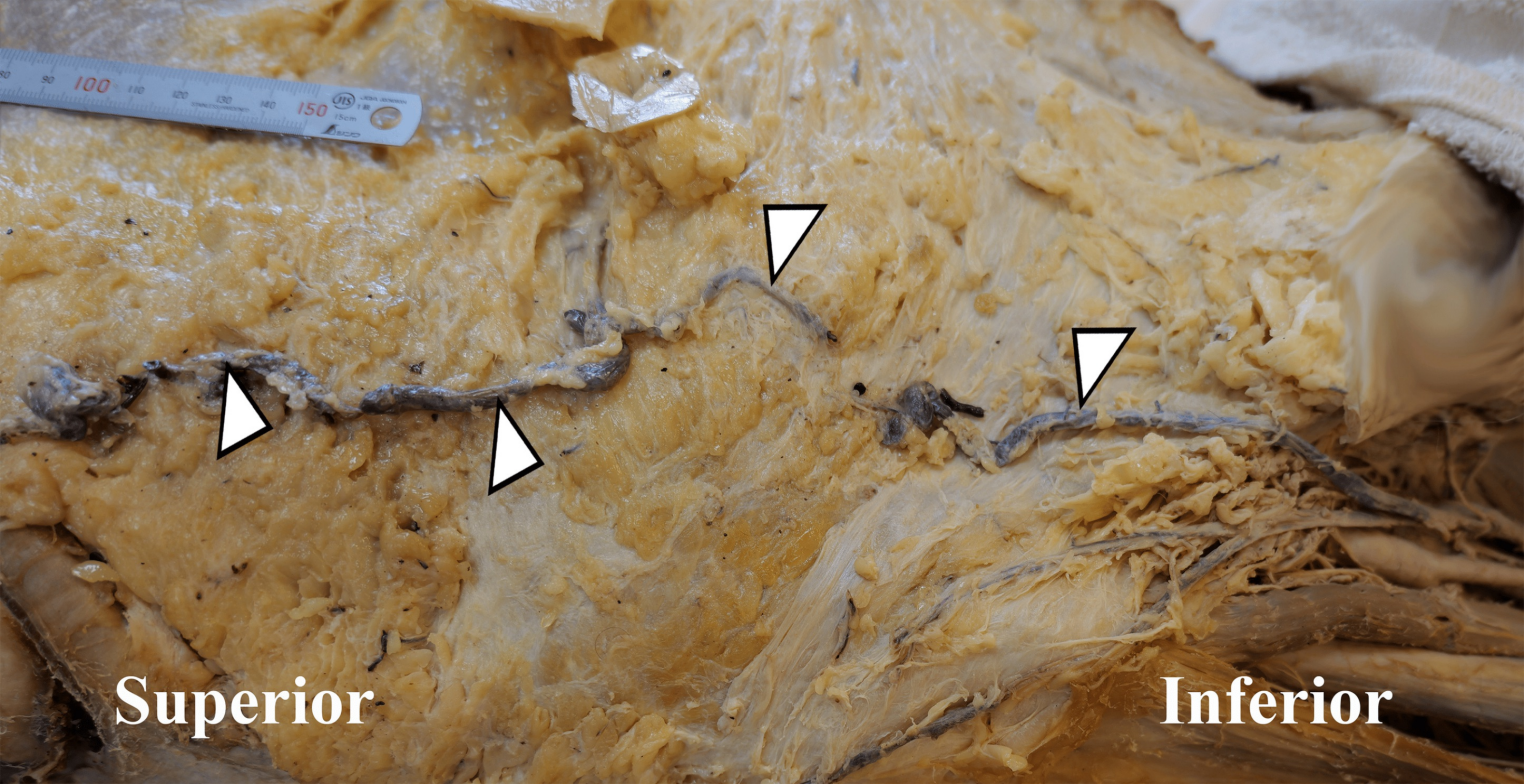
Lateral aspect of the right abdominal wall. Dilated right thoracic-epigastric vein (arrowheads) within the superficial fascia.

Upon completion of the dissection, the SVC was observed to be narrowed in diameter and was completely filled with fibrous material roughly 2 cm proximal to the heart. No lumen was visualized, so it was assumed to be 100% occluded proximal to the opening of the azygos vein (Figure 1), establishing the diagnosis of SVCS. Multiple mediastinal tumors were present, most notably directly posterior to the SVC, left brachiocephalic vein, and right lung. Venous collateral formation and vessel dilation were observed across the azygos system, the right internal thoracic vein, and several superficial vessels (Figure 2). The right superficial epigastric vein was enlarged, along with both the left and right inferior epigastric veins and the right thoracic-epigastric vein (Figure 3). Bilateral collateral veins were noted to connect to the internal thoracic vein superior to the first rib and run along the inferolateral costal groove of the fourth rib (Figure 2). Intercostal veins were grossly enlarged at all thoracic vertebral levels (Figure 2).

**Figure 4:**
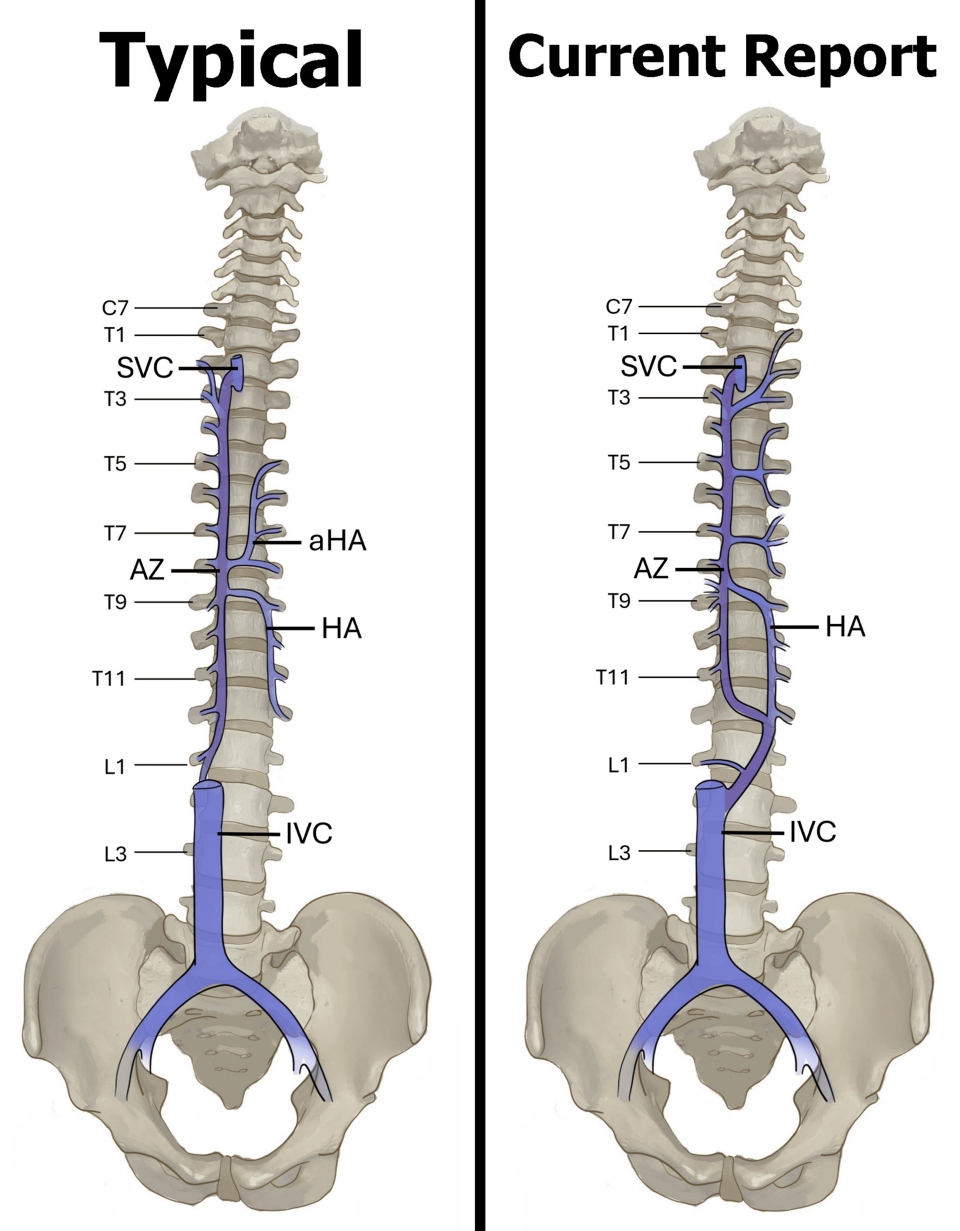
Diagrammatic sketch of the azygos venous system observed in normal anatomy (left) and the present study (right). SVC: superior vena cava; AZ: azygos vein; HA: hemiazygos; IVC: inferior vena cava; aHA: accessory hemiazygos Image Credit: Natalie Yoshioka, PhD

The azygos system morphology also reflected a potential SVCS compensatory mechanism (see Figure 4 for full azygos mapping). The azygos vein was dilated and descended from the SVC along the right aspect of the vertebral column down to the level of the vertebral body of T12. The azygos vein had a left anatomical direction across the T12 vertebral body to communicate with the hemiazygos vein instead of draining into the IVC. The azygos vein received drainage from the right T3-T11 posterior intercostal veins with two small additional accessory veins draining at the level of the T9 vertebral body. On the left side, four main branches crossed the vertebral bodies to drain into the azygos vein, one being a superior communicator with the hemiazygos vein. The left T1-T3 posterior intercostal veins drained into a common trunk that crossed the vertebral column and communicated with the azygos vein at the T3-T4 intervertebral disc level. The left T4-T6 posterior intercostal veins drained into a common trunk that crossed the vertebral column and had communication with the azygos vein at the T5-T6 intervertebral disc level. Lastly, the left T6-T8 posterior intercostal veins also drained into a common trunk that crossed the vertebral column and communicated with the azygos vein at the T7-T8 intervertebral disc level. Two intercostal veins drained from the left T6 vertebral level.

The hemiazygos vein had two communications with the azygos vein: superiorly, the hemiazygos vein crossed over the T8-T9 intervertebral disc to communicate with the azygos vein, and inferiorly, the azygos vein crossed right to left to the hemiazygos vein at the T12 vertebral level. The hemiazygos vein received drainage from the right T9-T12 and left T12 posterior intercostal veins before forming a communication, in combination with the azygos vein, with the IVC at the L2 level.

## Discussion

SVCS is estimated to occur in the range of one in 650 to one in 3,100 patients, with approximately 15,000 new cases identified each year in the United States [14]. SCVS saw a dramatic decrease in incidence during the 20th century, and today, most cases are the direct result of mediastinal malignancies or venous devices [15]. Typically, SVCS diagnosis is largely based on the patient’s history and presenting physical exam, with symptoms directly related to the degree of occlusion [2,14]. The most common presenting symptoms of SVCS include face/neck swelling combined with upper extremity edema, distended neck veins, and distended chest vein collaterals [2,14]. In our case, the common presenting symptoms were not observed, possibly due to the embalmment processes.

SVCS classification is traditionally based on anatomical location and occlusion percentage [10,15]. The location of the SVC occlusion is critical in treatment plans. If the occlusion is proximal to the azygos vein, the venous flow within the azygos vein will be directed retrograde into the IVC. If the obstruction is distal to the azygos vein, the venous flow will be anterograde into the SVC [5]. The direction of venous flow has a direct impact on treatment options [5]. There are currently two widely used classification systems: the Stanford classification and the newly proposed method by Azizi et al. [10,13,15]. In our case, we would classify this SVC as a Stanford Type IV: complete obstruction of the SVC and azygos venous system with the development of chest-wall and internal mammary venous collaterals, or as Azizi et al.’s Type IV, Class C: 100% infra-azygos venous occlusion [10,13,15].

The azygos system serves as the most important anastomosis between the SVC and IVC [13]. Azygos collateralization can also be classified under previous descriptions by Anson and McVay. The Anson and McVay system separates the azygos venous system into three types: type one is two vertically running (inferior to superior) azygos venous lines without connections between them, type two is two vertical azygos venous lines with at least one and up to five connections between them, and type three is a single vein in the middle that drains posterior intercostal veins from both sides [8]. Type two is the most encountered classification and is the closest fit with the previously described azygos collateralization. Knowledge of these different classifications and potential variations is important for treatment assessment; abnormal variations within the azygos system can be easily confused with an aneurysm, lymphadenopathy, or other abnormalities [8].

Treatment options for SVCS typically encompass endovascular techniques, such as balloon angioplasty and stenting, as well as radiation therapy, and occasionally open surgical intervention [3,4,13,15,16]. Knowledge of occlusion location, severity, and potential collateral formation aids in safer and more effective use of endovascular techniques, representing a shift toward minimally invasive approaches in managing SVCS [3,4,13,15-17]. It is unknown if this case received either a diagnosis or intervention for the postmortem observed SVCS.

## Conclusions

The incidence of SVCS had previously decreased due to advancements in managing intrathoracic malignancies. However, a recent surge in cases has been attributed to the increased use of thoracic implantable devices. As the incidence of SVCS increases, documentation gathered from postmortem findings can be used to more accurately predict venous remodeling in living patients and refine diagnostic approaches and therapeutic options.
